# The practice and effect of multi-disciplinary cooperative mobile devices in improving the satisfaction of inpatients on drug education

**DOI:** 10.4314/ahs.v24i4.48

**Published:** 2024-12

**Authors:** Shuangai Zhu, Chen Huang, Xiaoyun Hu, Yulan Xu, Lili Cheng, Hongying Pan

**Affiliations:** Sir Run Run Shaw Hospital, School of Medicine, Zhejiang University, Hangzhou, 310016 Zhejiang, China

**Keywords:** Multidisciplinary collaboration, mobile devices, drug education, patient satisfaction

## Abstract

**Objective:**

To explore the effect of multi-disciplinary cooperative mobile device-assisted drug education on the satisfaction of inpatients drug education.

**Methods:**

Mobile nursing vehicle, IPAD, and mobile phone were used to scan QR code to play drug propaganda videos and distribute drug propaganda materials. Doctors, nurses and pharmacists cooperated to complete drug education for inpatients. The satisfaction on drug education of inpatients was collected through a third-party satisfaction survey.

**Results:**

Before implementation, the four satisfactions of communicating about the drug, understanding the purpose of the drug, informing the role of the drug and informing the side effect of the drug were 83.9%, 80.4%, 83.8% and 83.9%, respectively, and after the implementation increased to 88.2%, 86.4%, 89.6% and 87.3% (P<0.05). The effective rate of drug education among inpatients increased from 95% before the implementation to 99.4%, and the medication compliance of inpatients increased from 93.1% before the implementation to 98.75%.

**Conclusion:**

Multi-subject cooperative mobile device-assisted drug education can improve the satisfaction of inpatients drug education, the efficiency of drug education and drug compliance.

## Introduction

Health education is a common demand for inpatients 1. Education of patients and their families is to help patients better participate in the treatment process and make reasonable treatment decisions. High-quality health education is an effective method and approach to improve the quality of medical services 2. Medication management is an independent or team-based pharmacy service that is patient-centered and an important service aimed at optimizing patient medication outcomes 3.

Drug therapy plays a crucial role in the treatment. Nurses are both the implementers of drug treatment and the guardians of safe drug use. Drug education is the planned provision of information, guidelines, resources, and skills relevant to the use of drugs. Nurses need to educate patients about drugs when executing the doctor's orders, help patients identify the side effects of drugs, educate the precautions for taking drugs, and increase the compliance and safety of taking drugs. However, due to the uneven health education ability of nurses, insufficient health education tools, single education method and mere formality, patients' satisfaction on drug education is generally low 4. It is particularly important to use a variety of educational resources to improve the effect of drug education for multi-disciplinary cooperation to participate in drug education.

The 2017 third-party satisfaction survey shows that our hospital has low scores in the four items of communicating about drugs, understanding the purpose of medication, informing the role of drugs, and informing about the side effects of drugs. The cooperative mobile device-assisted drug education has improved patients' satisfaction with the drug education, and the implementation effect is good.

## Methods

### Baseline characteristics

The inpatients from January 1, 2018 to December 31, 2018 are the research subjects. Survey method: Using the two-dimensional code of the Chinese version of HCAHPS provided by PG Company, our volunteers asked 20 discharged patients from each nursing unit to fill in a questionnaire every month to collect patient satisfaction data. Statistical methods: SPSS 22.0 statistical software was used to organize, count and analyse the data. The count data were expressed as percentages (%), and chi-square test and t test were used. P<0.05 was considered statistically significant.

### Multidisciplinary cooperation

The drug education of patients is completed by the multi-disciplinary cooperation of the doctor, the responsible nurse, and the clinical pharmacist in charge. The doctor in charge introduces the purpose and necessity of medication to patients during ward rounds. Clinical pharmacists participate in clinical work in ICU, cardiology, gastroenterology, oncology and other departments to assist doctors in choosing the best treatment plan 5-7. After the nurse in charge checks the drug order issued by the doctor, the special drug can be scanned with an iPad, nursing care car or mobile phone. The content of education is vivid and intuitive, and the content of medicine education is standardized. During common intravenous, subcutaneous, intramuscular, and oral administration, nurses educate patients or their families about drug effects, side effects, and precautions for use. The pharmacist completes the publicity and education of the patient's discharge from the hospital, providing professional medication guidance for the patient and their family members, informing the patient of the usage, dosage, best taking time, possible drug interactions, adverse reactions and precautions, so as to improve the patient's understanding of the drug.

### Mobile device assist

Each nursing unit in the hospital is equipped with 4-5 mobile nursing vehicles, of which each bed in the orthopaedic nursing unit is provided with an iPad. They can also use the patient's smartphone to scan the code through WeChat, and use mobile devices to assist in drug education, so that patients can learn at any time. Teach-back 8 was used to evaluate the effect of drug education after drug education. Teach-back is recognized and recommended by the National Quality Forum in the United States as a simple and effective health education method 9. Teach-back is to let the patients express their understanding of the content of the drug education in their own language after the drug education is given to the patient. For the information that the patient misunderstands or does not understand, the nurse will teach again until the patient has all the information correctly.

### Production of video and mission materials

The hospital has strict requirements on the production of drug propaganda videos and propaganda materials. Each specialty conducts a needs survey, and produces educational videos and educational manuals according to the needs of patients. The hospital's propaganda video requires professionals to repeatedly revise and verify the written materials and screen design before shooting, and the shooting samples are submitted to the professionals for review, and can only be used after the review. Drug propaganda materials should be easy to understand, with pictures and texts. Drug propaganda materials should be reviewed by head nurses and specialists to ensure the accuracy of the content. They should be submitted to the hospital health education committee for review, and then typesetting and printing after approval. At present, our hospital's drug education videos include aerosolized drug inhalation education, standardized insulin injection, insulin dosage forms and precautions for use, and the use of Goserelin. Drug education materials include warfarin, low molecular weight heparin, insulin, etc.

### Employee training

The mastery of medical staff's drug knowledge will affect the effect of drug education. In order to improve the drug knowledge of medical staff, the hospital has taken a series of measures: after new employees are admitted to the hospital, the whole hospital will have drug knowledge training; Within 18 months, nurses will have a pharmacology knowledge assessment to urge nurses to learn drug knowledge independently; The department would list common drugs and special drugs, including drug names, functions, side effects, precautions for use, etc., for nurses in the department to consult at any time; Invite clinical pharmacists to give lectures on drug-related content to medical staff in the whole hospital; Discussion on the case of serious adverse drug reactions in the hospital. The hospital sets up a full-time education coordinator, and each department selects part-time health educators to train medical staff on the basic theory of education to ensure that the educators have good educational ability 7.

### Rational drug use monitoring system

The rational drug use monitoring system is related to the hospital electronic information system. The system is based on the basic characteristics and requirements of clinical rational drug use.

It is necessary to use information technology to carry out structured processing of pharmaceutical knowledge, which can realize automatic review of medical orders and online query of medical information, help clinical professionals to grasp drug knowledge in a timely and effective manner in the process of drug use, prevent the occurrence of adverse drug events, and promote clinical rational drug use. Medical staff can log in to the system and select a patient to query the patient's drug orders. By right-click to select a drug, the drug instruction manual will appear, and drug information can be browsed. The drug instructions in the rational drug use monitoring system are complete, including information on more than 80,000 drugs. The system includes drug names, ingredients, properties, indications, specifications, usage and dosage, adverse reactions, contraindications, precautions, drug interactions, etc. When the clinical medical staff have questions about the drug information, they can consult it in time, which provides convenience for the medical staff. In the electronic information system, a doctor's order is set to associate food and drug interactions. When a doctor issues a drug order, if there is a food taboo, there will be a reminder in the doctor's order, and a black light will be on for absolute taboos. Limitation of high warning drug doses, enhanced CT focus on checking renal function, if not detected, the system recommends renal function tests, etc., to avoid the occurrence of unexpected events and ensure the safety of patients' medication.

### Monitoring indicators

Our hospital adopts the Hospital Consumer Assessment of Healthcare Providers and Systems Survey (HCAHPS) conducted by Press Ganey (PG) in the United States, which was conducted by the Center for Medicare and Medicaid Services (CMS) in 2002. Initiated jointly with the Administration of Health Care and Quality (AHRQ), it is the most comprehensive patient satisfaction assessment in the United States. It is used by major well-known hospitals such as Mayo, Massachusetts General Hospital, and Johns Hopkins Hospital in the United States. It is a comprehensive satisfaction assessment independently operated by a third-party research company. HCAHPS asks patients to evaluate the items listed in “Never, Sometimes, Usually and Always”. The items about drugs include: communicating about the drug, understanding the purpose of the drug, telling the effect of the drug, and telling the side effect of the drug. Data collection method: Using the two-dimensional code of the Chinese version of HCAHPS provided by PG company, college student volunteers asked 20 discharged patients from each nursing unit to fill in the questionnaire every month, and directly uploaded to the PG database after completion. Reports are presented monthly, quarterly, and annually.

### Quality control

The Nursing Department regularly conducts nursing quality inspections on nursing units, and effectively controls the quality of drug education. Nurses need to record a health education record after giving drug education to patients, and the results of drug education need to be evaluated. When patients partially master the content of the education, they need to conduct drug education and record again to ensure the effectiveness and continuity of drug education. During the nursing quality inspection, problems are found and reported to the unit head nurse in a timely manner. The nursing unit makes rectifications according to the existing problems. The hospital head nurses will give feedback on drug problems common to the whole hospital and implement improvement measures.

## Results

Through a third-party satisfaction survey, the drug education satisfaction, drug education effectiveness and medication compliance of inpatients in 2019 were collected. Using SPSS 22.0 software for analysis, by the chi-square method, we defined the P value <0.05 as significant statistical significance.

Before the implementation, they communicated about the drug, understood the purpose of the drug, informed the role of the drug, and informed the side effects of the drug. The satisfaction rates of these four items were 83.9%, 80.4%, 83.8%, and 83.9%, respectively. After the implementation, the satisfaction of these four items increased to 88.2%, 86.4%, 89.6%, 87.3%. The difference was statistically significant (Table 1).

**Table 1:** Satisfaction of inpatients with drug education

Before implementation (%)		Select number of patients/number of surveys	After implementation (%)	Select number of patients/number of surveys	Chi-square value	P value
Communicate about medicines	83.9	3864/4606	88.2	2784/3156	28.264	<0.001
Understand the purpose of medication	80.4	5472/6803	86.4	4302/4980	71.938	<0.001
Inform the role of the drug	83.8	3837/4600	89.6	2828/3155	59.984	<0.001
Inform about the side effects of the drug	83.9	3865/4605	87.3	2756/3156	17.226	<0.001

The drug knowledge awareness of inpatients was collected through one-to-one patient consultation, and a drug knowledge awareness survey was conducted among 160 inpatients, and the drug knowledge awareness was divided into complete knowledge, partial knowledge, and no knowledge in Table 2.

**Table 2:** Awareness survey of drug knowledge among hospitalized patients, n (%)

	Number of cases	Fully aware	Partially aware	Don't know
Before implementation	160	152 (95)	6 (3.75)	2 (1.25)
After implementation	160	159 (99.4)	1 (0.6)	0 (0)

The data on medication compliance of inpatients was collected through the quality control inspection of the Nursing Department, and 160 inpatients were surveyed on the medication compliance, and the medication compliance was divided into good, medium and poor (Table 3).

**Table 3:** Inpatient medication compliance survey, n (%)

	Number of Cases	Good	Moderate	Poor
Before implementation	160	149 (93.1)	7 (4.4)	4 (2.5)
After implementation	160	158(98.75)	2(1.25)	0(0)

## Discussion

After the implementation of multi-disciplinary cooperation in mobile device-assisted drug education, we communicated about drugs, understood the purpose of drug use, informed about the role of drugs, and informed about the side effects of drugs. The satisfaction of these four items has been significantly improved. Drug education programs focused on providing information about the negative consequences of drugs and changing the attitudes and behaviours of patients in relation to drug use. The drug education model assisted by the multi-disciplinary cooperative mobile device of the doctor in charge, the responsible nurse, and the clinical pharmacist can improve the satisfaction of patients' drug education. A good doctor-patient relationship is the premise of health education. The drug education model of multi-disciplinary cooperation is better than the ordinary drug education. The drug education model of multi-disciplinary cooperation promotes communication among medical staff, reflects humanistic care in the process of drug education, makes patients and their families feel that medical staff attach great importance to them, and enhances the trust among doctors, nurses, and patients. The drug education model of multi-disciplinary cooperation respects the individual learning needs and learning types of patients, appropriately selects educational tools, provides high-quality drug education for patients, promotes patients' mastery of knowledge, and improves patients' satisfaction with drug education.

Traditional health education is mainly based on simple knowledge instillation by preaching or distributing brochures. Studies have shown that after drug education, 40%-80% of the information is forgotten or nearly 50% of the information is misunderstood 10. Teach-back is a two-way information transmission mode. Health education is not terminated after the transmission of information, but it is necessary to further evaluate the patient's understanding and mastery of the information, allowing patients to repeat and demonstrate the content of the education in their own language, and evaluate the effect of education in a timely manner 11. In Teach-back, nurses use easy-to-understand language in the process of transmitting information, which not only allows patients to learn and master the content of education, but also allows patients to fully feel the care and respect of nurses in the process of education. Previous studies have suggested that the form of Teach-back education can improve the self-management ability of patients and reduce the readmission rate 12-13. Through the drug education assisted by mobile devices, the video content is intuitive, vivid, and easy to understand, which is conducive to improving patients' understanding, stimulating learning enthusiasm, enabling patients to cooperate with treatment, and increasing patients' compliance with medication 14-15. Provide a variety of drug education materials according to the needs of patients, and the content of the education and education is standardized and homogeneous, which improves the efficiency of drug education and education.

Rational drug use monitoring system software has become an indispensable tool in hospital pharmacy information management. It realizes the information management of drug safety supervision process, improves the level of rational drug use, and ensures patient drug safety 16. The rational drug use monitoring system is convenient, fast and practical, which provides convenience for medical staff to access drug information and provides guidance for clinical drug use. The use of the rational drug use monitoring system improves the medical staff's mastery of drug knowledge 17, enhances their clinical professional drug use skills, and pharmacology knowledge 18 to improve the ability of drug safety review and control, and to escort the drug safety of clinical patients. Rational drug use monitoring system software needs to be regularly maintained and updated to meet clinical needs.

There were several limitations in our study. First, the sample size in our study was not large, the future studies should include more samples to validate our results. Second, there was no long-term follow-up in our study. Third, our study was based on the data form a single center, a multicentre study should be performed. Last, different types of drugs should be analyzed separately; however, we were not allowed to perform such analysis due to the limitation on the data we got.

The multi-disciplinary cooperative mobile device-assisted drug education model can improve the satisfaction of patients in drug education and improve the compliance of patients with taking medication. The implementation of this project has improved the drug knowledge and education ability of medical staff. Drug education videos and education materials need to be updated regularly. We will further increase the types of drug education videos to meet the needs of different types of patients in the future. The clinical implementation effect of multi-subject cooperative mobile device-assisted drug education is worthy of reference and promotion.

## References

[1] Lu Renyu, Peng Jun, Wang Xiaohui (2000). Investigation on health education needs of inpatients [J]. Chinese Journal of Hospital Management.

[2] Yang Lili, Feng Jin, Lou Qingqing (2004). The role of health education coordinator in medical service [J]. Chinese Journal of Nursing.

[3] Deepak S, Dharun T, Bhuvana K P Preparation and Characterization of Composite Fabric for Soft Body Armours. International Conference on Advancements in Polymeric Materials.

[4] Hu Rong (2002). Analysis and countermeasures of problems in clinical health education [J]. Chinese Journal of Nursing.

[5] Ma Ke (2010). Learning from foreign experience and creating clinical pharmacy with Chinese characteristics—Introduction to clinical pharmacy in Run Run Shaw Hospital Affiliated to Zhejiang University School of Medicine [J].

[6] Friedman JA, Cosby R, Boyko S (2011). Effective teaching strategies and methods of delivery for patient education: a systematic renew and practice guideline recommendations[J]. Journal of Cancer Education.

[7] Shen Liying, Yang Lili, Xu Yulan (2011). Implementing patient health education through multidisciplinary cooperation [J]. Chinese Journal of Hospital Management.

[8] Schillinger D, Piette J, Grumbach K (2003). Closing the loop: physician communication with diabetic patients wbo bave low heahh literacy[J]. Arch Intern Med.

[9] Peter I, Robinson P, Jordan M (2015). Reducing read—missions using leach—back: enhancing patient and family education[J]. J Nurs Adm.

[10] Farris C (2015). The teach back method[J]. Home Healthc Now.

[11] Pan Cuiliu, Zhang Shuang, Zhang Zhiru (2016). Application status of teaching back method in nursing health education [J]. Journal of Nursing.

[12] Peter D, Robinson P, Jordan M (2015). Reducing readmissions using teach-back: enhancing patient and family education[J]. J Nurs Adm.

[13] Dantic DE (2014). A critical review of the effectiveness of ‘teach- back’ technique in teaching COPD patients self-management using respiratory inhalers[J]. Health Educ J.

[14] Yang Chi, Rigorous, Shen Ting (2016). Application of video health education in patients with spinal surgery[J]. Journal of Nursing.

[15] Kandula NR, Malli T, Zei CP (2011). Literacy and retention of information after a multimedia diabetes education program and teach-back[J]. Health Commun.

[16] Gao Dewei, Xu Lining, Li Tianzhi (2017). Exploration on the comprehensive management mode of geriatric surgery in the perioperative period [J]. Chinese Journal of Health Medicine.

[17] Wei Hua, Song Jiangyan, He Xiaolan (2016). Investigation on the research status of stratified training of clinical nurses in my country [J]. China Nursing Management.

[18] Gao Yanling, Zhong Xiujuan, Xue Mei (2016). Analysis on the construction effect of rational drug use monitoring system in our hospital [J]. China Pharmacy.

